# RhoA Is Essential for Maintaining Normal Megakaryocyte Ploidy and Platelet Generation

**DOI:** 10.1371/journal.pone.0069315

**Published:** 2013-07-23

**Authors:** Aae Suzuki, Jae-Won Shin, Yuhuan Wang, Sang H. Min, Morty Poncz, John K. Choi, Dennis E. Discher, Chris L. Carpenter, Lurong Lian, Liang Zhao, Yangfeng Wang, Charles S. Abrams

**Affiliations:** 1 Department of Hematology/Oncology, School of Medicine, University of Pennsylvania, Philadelphia, Pennsylvania, United States of America; 2 Pharmacology Medicine, School of Medicine, University of Pennsylvania, Philadelphia, Pennsylvania, United States of America; 3 Division of Hematology, Children's Hospital of Philadelphia, Philadelphia, Pennsylvania, United States of America; 4 Hematopathology, St. Jude Children’s Research Hospital, Memphis, Tennessee, United States of America; 5 Clinical Oncology, GlaxoSmithKline, Philadelphia, Pennsylvania, United States of America; University of Leuven, Belgium

## Abstract

RhoA plays a multifaceted role in platelet biology. During platelet development, RhoA has been proposed to regulate endomitosis, proplatelet formation, and platelet release, in addition to having a role in platelet activation. These processes were previously studied using pharmacological inhibitors *in vitro*, which have potential drawbacks, such as non-specific inhibition or incomplete disruption of the intended target proteins. Therefore, we developed a conditional knockout mouse model utilizing the CRE-LOX strategy to ablate RhoA, specifically in megakaryocytes and in platelets to determine its role in platelet development. We demonstrated that deleting RhoA in megakaryocytes *in vivo* resulted in significant macrothrombocytopenia. RhoA-null megakaryocytes were larger, had higher mean ploidy, and exhibited stiff membranes with micropipette aspiration. However, in contrast to the results observed in experiments relying upon pharmacologic inhibitors, we did not observe any defects in proplatelet formation in megakaryocytes lacking RhoA. Infused RhoA-null megakaryocytes rapidly released platelets, but platelet levels rapidly plummeted within several hours. Our evidence supports the hypothesis that changes in membrane rheology caused infused RhoA-null megakaryocytes to prematurely release aberrant platelets that were unstable. These platelets were cleared quickly from circulation, which led to the macrothrombocytopenia. These observations demonstrate that RhoA is critical for maintaining normal megakaryocyte development and the production of normal platelets.

## Introduction

The small GTPase RhoA is an intracellular signaling protein that regulates actin cytoskeletal dynamics necessary for cell motility and stress fiber formation [1], [2]. During platelet development, RhoA is speculated to control endomitosis and proplatelet formation [3]–[5]. Furthermore, in platelets, RhoA is involved in directing cytoskeletal reassembly to facilitate shape change and granule release during hemostasis [6]–[8].

**Figure 1:**
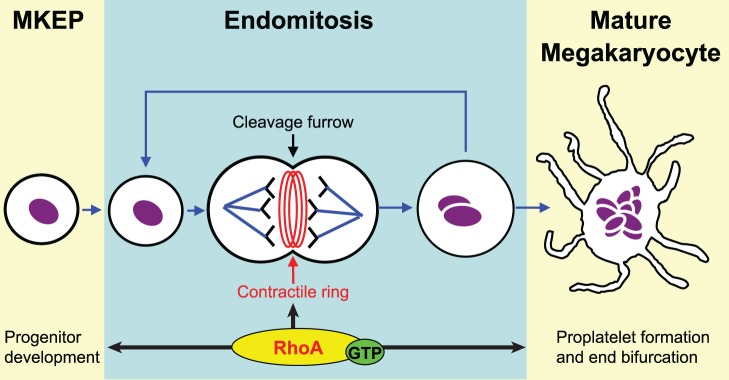
RhoA is essential for two stages of platelet production. RhoA coordinates cytokinesis of promegakaryocytes and endomitosis of megakaryocytes by regulating effectors that control the actin contractile ring. The contractile ring underlies and constricts the cleavage furrow, which facilitates cell division. Another potential site of regulation is the ROCK-myosin pathway during thrombopoiesis. Actomyosin forces limit proplatelet formation, which ultimately controls platelet size.

Developing megakaryocytes undergo significant changes in morphology, which is driven by RhoA. They enter several cycles of endomitosis that lead to their characteristic enlarged polyploidy phenotype (Figure 1) [9]. After DNA duplication, the actin-myosin contractile ring forms around the equator of the cell bisecting the mitotic spindle and serves as a scaffold for the developing cleavage furrow where the cell would normally segregate during cytokinesis. In most cells, RhoA facilitates the assembly of the contractile ring by polymerizing actin filaments and by activating myosin through Rho kinase (ROCK) [10]. However, because of the unique biology of megakaryocytes, guanine exchange factors (GEFs) are down-regulated in endomitosis. This leads to the deactivation of RhoA, which causes contractile ring disassembly and cleavage furrow regression, which thereby aborts cell division resulting in the multinucleated morphology of megakaryocytes [5], [11].

RhoA has also been postulated to regulate thrombopoiesis in mature megakaryocytes by controlling actin cytoskeletal forces [12]. Though microtubule elongation has been implicated as the primary force in proplatelet formation, in cultured megakaryocytes, expression of a constitutively active form of RhoA decreases proplatelet length, presumably by preventing the unfolding of pseudopodial extensions from demarcation membranes [3].

Studies that have developed the current models of RhoA involvement in megakaryopoiesis have relied on the use of prolonged incubation *in vitro* with pharmacological toxins such as C3 ADP-ribosyltransferase. However, these inhibitors may nonspecifically deactivate other members of the Rho subfamily such as RhoB/C, Rac1, or CDC42. Additionally, it is also unclear as to the completeness of this RhoA disruption [13]. To address these issues, Pleines, et al., have generated transgenic mice with megakaryocyte/platelet-specific deletion of RhoA [8]. These mice exhibited platelets that have mild functional deficits in shape change, granule secretion, and clot retraction. Interestingly, these mice lacking RhoA in their megakaryocytes and platelets also developed macrothrombocytopenia.

To further understand the role of RhoA in endomitosis and in thrombopoiesis during megakaryocyte development, we independently generated a transgenic mouse model in which RhoA is completely deleted in only megakaryocytes and in platelets. We confirmed the macrothrombocytopenia and examined the effect of this RhoA deficiency on megakaryopoiesis. We also tested the role of RhoA in megakaryocyte and platelet biology and found a role for RhoA in the survival of both megakaryocytes and platelets. We also found that RhoA null megakaryocytes had a defect in their membrane rheology. Finally, in contrast to previous findings, genetic ablation of RhoA did not increase proplatelet formation.

## Methods

### Animals

**Figure 2:**
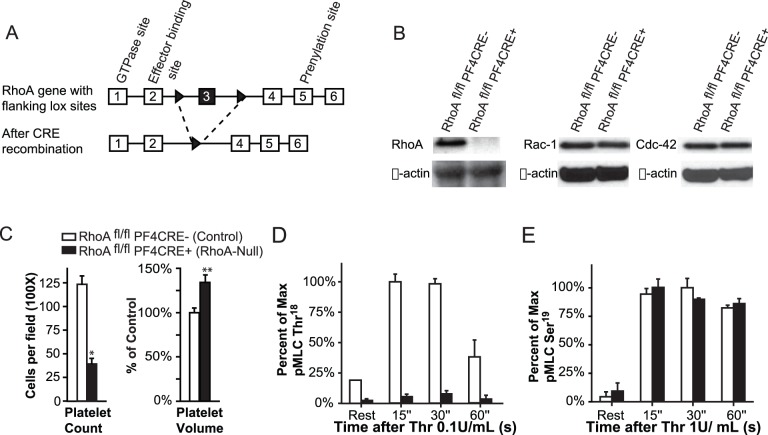
Mice with targeted deletion of RhoA in megakaryocytes and in platelets exhibit macrothrombocytopenia and impaired MLC phosphorylation. (A) The RhoA transgene construct contains loxP sites flanking exon three. These mice were crossed with mice expressing PF4 promoter-driven CRE recombinase. (B) Western blotting confirmed that platelets from RhoA^fl/fl^ PF4CRE^+^ positive mice did not express detectable RhoA protein, but had normal amounts of Rac1 and CDC42. (C) Cell blood counts were normal, except that the RhoA^fl/fl^ PF4CRE^+^ mice were macrothrombocytopenic. * indicates p<0.005, ** indicates p<0.05. The immunoblot of thrombin-treated platelets were probed with antibodies against the phospho-MLC2 Thr^18^ (D) or Ser^19^ (E). Phosphorylation of the MLC2 Ser^19^ residue was normal (D), but phosphorylation of the MLC2 Thr^18^ residue was undetectable in RhoA-null platelets for all stimulation times (E).

This study was carried out in strict accordance with the recommendations in the Guide for the Care and Use of Laboratory Animals of the National Institutes of Health and approved by the Institutional Animal Care and Use Committee (IACUC) of the University of Pennsylvania. All mice were maintained in the animal facility of the University of Pennsylvania in accordance with National Institutes of Health guidelines and under IACUC–approved animal protocols (705465). To produce mice that were lacking RhoA in megakaryocytes and in platelets, a homozygous floxed RhoA (RhoA^fl/fl^) mouse line was first generated. The LoxP sites flanked the third exon of the RhoA gene (Figure 2A). This exon encodes the P-loop and switch I domains, which confer binding to RhoA regulators and effectors [14], [15]. These mice were crossed with a mouse line that expressed CRE recombinase under a PF4 promoter (the PF4CRE^+^ mouse line was a generous gift from Radek Skoda, of the University of Basel, Switzerland) [16]. Complete blood counts (CBCs) and mean platelet volumes were performed by using the Drew Hemavet Hemacytometer (HV1700). Platelet counts were also performed manually from peripheral blood smears stained with hematoxylin/eosin (H/E).

For the megakaryocyte infusion experiments, we used transgenic mice that were lacking mouse (m) αIIb, but expressing human (h) αIIb in their platelets, which allows us to distinguish between donor mouse (mαIIb^+/+^/hαIIb^−/−^) and recipient mouse (mαIIb^−/−/^hαIIb^+/+^) as previously described [17].

### Immunoblotting

Washed platelet preparation and immunoblotting procedure has been described previously [18]. The following primary antibodies were used: RhoA (Cytoskeleton, Inc), Rac1 (Santa Cruz), CDC42 (Santa Cruz), and activated caspase-3 (Millipore). The blots were developed with ECL (GE Amersham). Films were scanned and were analyzed using Image J.

### Megakaryocyte Population and Ploidy

For histological analysis, bone marrow and spleen from 8-week old mice were fixed in 4% paraformaldehyde, decalcified (Cal Ex; Fisher), and paraffin-embedded. Sections were stained with H/E. To measure the number of megakaryocytes, bone marrow was flushed from the femurs of 8–10 week old mice as previously described [19]. The cells were stained with CD41 (integrin αIIb) antibody conjugated with Alexa 488 (BD Sciences), and cells were counted on the FACS Calibur flow cytometer (BD Biosciences), discriminating for size to subtract out platelets. To determine the megakaryocyte ploidy, the cells were also stained with propidium iodide to determine the relative DNA content. FlowJo software was used to analyze all flow cytometry data.

### Megakaryocyte Fetal Liver Culture and Infusion

Megakaryocyte cultures were derived from fetal livers (E13.5) as described previously [20], [21]. After 4 days, cultured megakaryocytes were isolated by sedimentation through a 1.5%/3% BSA step gradient. For proplatelet counts, isolated megakaryocytes were allowed to grow in tissue culture plates for an additional day and imaged with an inverted microscope (Leica DM IRB). Megakaryocytes bearing proplatelets were counted from at least 40 fields per sample. For confocal images, isolated megakaryocytes were also allowed to grow for an additional day. The next day megakaryocytes were plated onto tissue culture plates with poly-L-lysine (Sigma) coated coverslips and spun down. These coverslips were stained for β1-tubulin (Abcam) and Alexa 494-phalloidin (Invitrogen). An anti-rabbit FITC secondary antibody (Invitrogen) was used to detect β1-tubulin antibody [21]. The slides were examined with a laser spinning disk confocal microscope (Nikon Eclipse Ti-U). For both of the microscopic analyses, genotypes of micrographs were randomized prior to analysis to avoid bias.

For megakaryocyte infusion, the cells were diluted to 2.5 million cells per 250 µL. For platelet infusion, washed platelets were prepared as previously described, and diluted to 250 million platelets per 250 µL [18]. After anesthetization with isoflurane, cell suspensions were injected into the retro-orbital sinuses of transgenic mice expressing the mαIIb^−/−/^hαIIb^+/+^ marker [17]. Blood was then collected from the retro-orbital sinuses for CBC and flow analysis at various time points. For flow analysis, cells were stained for the mαIIb and hαIIb epitopes to determine the relative platelet population over time by using species-specific anti-CD41 primary antibodies as described [17].

### *In vivo* Platelet Depletion

For the platelet depletion assay, mice were injected with 2 µg/g of GPIbα antibody (Emfret) into the retro-orbital sinuses [22]. Blood was collected from the retro-orbital sinuses for platelet counts that were analyzed either on a Hemavet devise or manually by examination of the peripheral blood smears.

### Cell Membrane Compliance Micropipette Analysis

This procedure was previously described [23]. First, the cultured megakaryocytes were stained with 7AAD and CD41 to aid in the identification of live megakaryocytes. Micropipettes were attached to a dual stage water manometer and aspirated megakaryocytes were then visualized under the microscope. Aspiration pressure was applied, and pressure was monitored with a transducer (Validyne). The length of the membrane deformation was measured over time, and membrane compliance (J(t)) was calculated as a function of aspiration pressure (kPa), length (L, µm) of extension, and diameter (D, µm) of the pipette at a given aspiration duration [24].

### Statistical Analysis

Student t-tests were performed for analysis of significance by using Excel (Microsoft).

## Results

To examine the role of RhoA small GTPase in megakaryopoiesis and in platelet formation, we generated mice specifically lacking this protein in their megakaryocytes and in their platelets. Platelets derived from RhoA^fl/fl^ PF4CRE^+^ mice did not express any detectable RhoA protein as measured by immunoblotting (Figure 2B). Importantly, loss of RhoA did not induce compensatory up-regulation of the closely related GTPase family members such as Rac1 and CDC42 (Figure 2B). The RhoA^fl/fl^ PF4CRE^+^ transgenic mice were grossly normal, and had body masses, organ morphologies, leukocyte counts, and hemoglobin levels that were indistinguishable from their littermate control, RhoA^fl/fl^ PF4CRE^−^ mice (data not shown). The mice grew up to adulthood without any observable increase in spontaneous hemorrhage, thrombosis, or mortality (data not shown). Conversely, RhoA^fl/fl^ PF4CRE^+^ mice displayed macrothrombocytopenia with a 66% reduction in platelet counts and a 25% increase in platelet volume as measured by automated platelet counts and confirmed by manual review of peripheral blood smears (Figure 2C). We performed various platelet functional assays such as aggregation, secretion, and platelet spreading experiments (data not shown), and found mild defects that were consistent with an independently derived RhoA^fl/fl^ PF4CRE^+^ mouse line observed by Pleines et al [8]. Similar to their findings, we also observed a defect in clot retraction (not shown.).

Previous studies have demonstrated that the RhoA effector, ROCK is important for MLC2 phosphorylation and consequently clot retraction [25]. We measured ROCK-mediated MLC2 phosphorylation in RhoA^fl/fl^ PF4CRE+ platelets after stimulation with thrombin. MLC2 can be phosphorylated at two sites. First, MLC2 can be phosphorylated at Thr^18^ by ROCK. Second, MLC2 can be phosphorylated at Ser^19^ by a RhoA independent pathway. As predicted, platelets lacking RhoA had normal phosphorylation of Ser^19^ (Figure 2E), but no phosphorylation of Thr^18^ (Figure 2D).

### Decreased RhoA-null Megakaryocytes Lead to Macrothrombocytopenia

**Figure 3:**
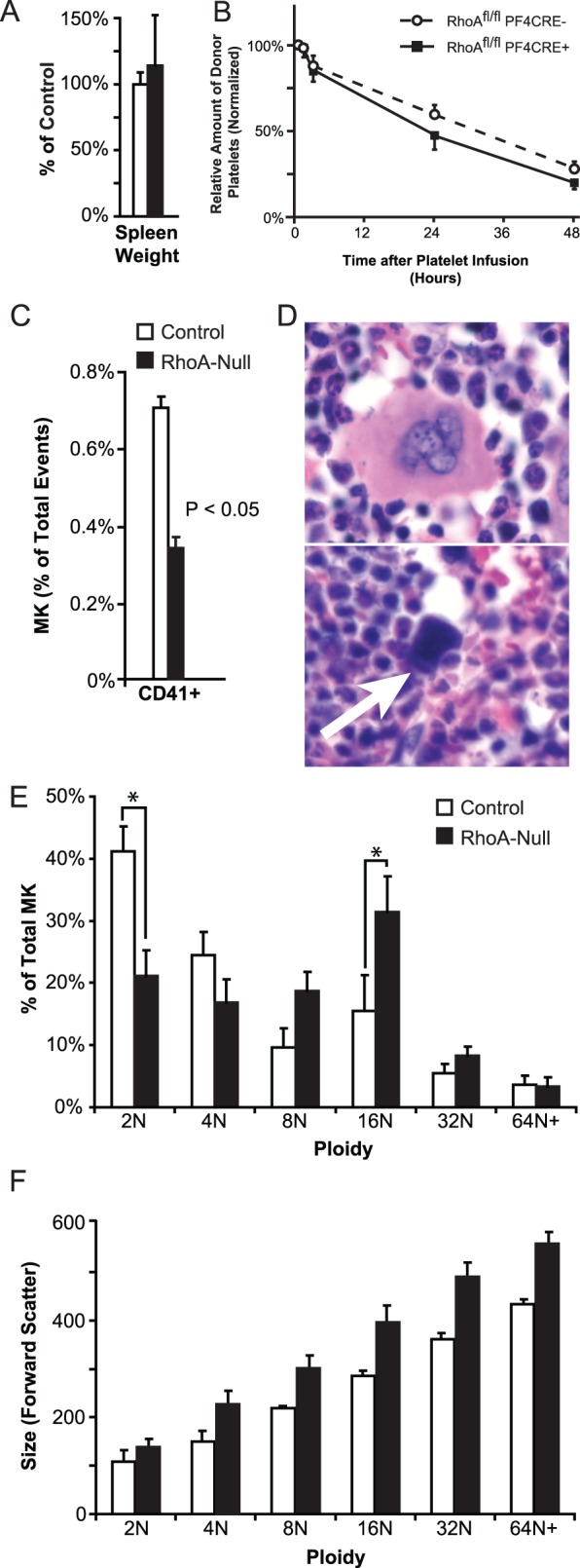
RhoA deletion disrupts megakaryocyte development. (A) The differences in spleen weights were not statistically significant. (B) When *adult* RhoA-null platelets were infused into mαIIb^−/−/^hαIIb^+/+^ mice, the time course of platelet survival was similar to that of their controls. Differences were not statistically significant. Shown is the mean ±SE (N = 3). Values were normalized to platelet levels at the initial draw for each experimental group (30 minutes after infusion). (C) Flow cytometry revealed that the megakaryocyte population, as a percentage of the total bone marrow cells, was diminished in the RhoA^fl/fl^ PF4CRE^+^mice. Megakaryocytes were discriminated by size and by the use of a FITC-labeled anti-CD41 antibody. Mean ± SE is shown. N = 3. (D) On the top is a representative image of a normal megakaryocyte, and on the bottom is a megakaryocyte (arrow) undergoing apoptosis that is exhibiting dark, pyknotic nuclei and scant cytoplasm. In the bone marrow and in the spleen of RhoA^fl/fl^ PF4CRE^+^ mice, megakaryocytes are more likely to exhibit this phenotype. Magnification is 40×. (E) The RhoA-null megakaryocyte population has a higher ploidy than normal. Ploidy was calculated by DNA staining with propidium iodide, and we measured staining intensity with flow cytometry. Mean ± SE is shown; * indicates P<0.05. N = 3. (F) Megakaryocyte size (forward scatter) was measured as a function of ploidy. The results show that RhoA-null megakaryocytes were larger than the control cells at all ploidy. Mean ± SE is shown; * indicates P<0.05; ** indicates P<0.01. N = 4.

To determine the cause of the thrombocytopenia, we measured spleen weight to test for splenic sequestration. RhoA^fl/fl^ PF4CRE^+^ mice had no statistically significant change in their spleen size compared to their littermate controls (right graph, Figure 3A). Next, we examined if platelet clearance caused thrombocytopenia by measuring platelet half-life. For these experiments, we used transgenic mice that expressed human αIIb^+/+^, but not mouse αIIb^+/+^, which allowed us to distinguish between donor and recipient platelets. After isolating washed platelets from the blood of the donor adult RhoA^fl/fl^ PF4CRE^+^ mice or the control mice, we injected the platelets into the retro-orbital sinuses of the recipient mαIIb^−/−/^hαIIb^+/+^ mice and measured platelet levels over time. We observed that there was no statistical difference in the RhoA-null platelet half-life using this method (Figure 3B).

Given that neither splenic sequestration nor decreased platelet half-life appeared to cause the thrombocytopenia, we reasoned that the thrombocytopenia was attributed to defective platelet production, and therefore analyzed the megakaryocytes. Megakaryocyte counts in the bone marrow of RhoA^fl/fl^ PF4CRE^+^ mice with flow cytometry showed a 51.5±4.2% reduction when compared with RhoA^fl/fl^ PF4CRE^−^ mice (Figure 3C). Apoptosis of the megakaryocytes was also higher in the RhoA^fl/fl^ PF4CRE^+^ mice (Figure 3D). Histological examination of the spleens derived from RhoA^fl/fl^ PF4CRE^+^ mice revealed that 31±10% of the RhoA-null megakaryocytes had pyknotic nuclei as compared to the megakaryocytes found in the littermate control mice (1.0±0.5%; P<0.05). Further morphologic analysis of the bone marrows by a pathologist blinded to the genotypes demonstrated that the megakaryocytes in the bone marrow of all RhoA^fl/fl^ PF4CRE^+^ samples had moderate to scant cytoplasm. In contrast, all of the control bone marrow samples had megakaryocytes with mostly abundant and normal appearing cytoplasm.

### RhoA is Required for Ploidy Distribution and Size of Megakaryocytes

Based upon these data, we postulated that the increase of megakaryocytes undergoing apoptosis might be due to the dysregulation of the contractile ring during endomitosis. To test this hypothesis, we measured cell ploidy and division. The contribution of RhoA to ploidy was determined by flow cytometry after staining DNA with propidium iodide and labeling CD41-positive cells. We observed that modal ploidy in control megakaryocytes was 2N with the distribution tapering off at increasing N (Figure 3E). In contrast, the modal ploidy in the RhoA-null megakaryocytes was 16N. Cultured megakaryocytes derived from fetal livers of 13.5 day-old embryos revealed that RhoA-null megakaryocytes were much larger than their controls at *every* ploidy tested (Figure 3F). Together, this supports the hypothesis that RhoA contributes to the regulation of endomitosis [4], [5].

### Membrane Rheology of CD41-labelled Megakaryocytes

**Figure 4:**
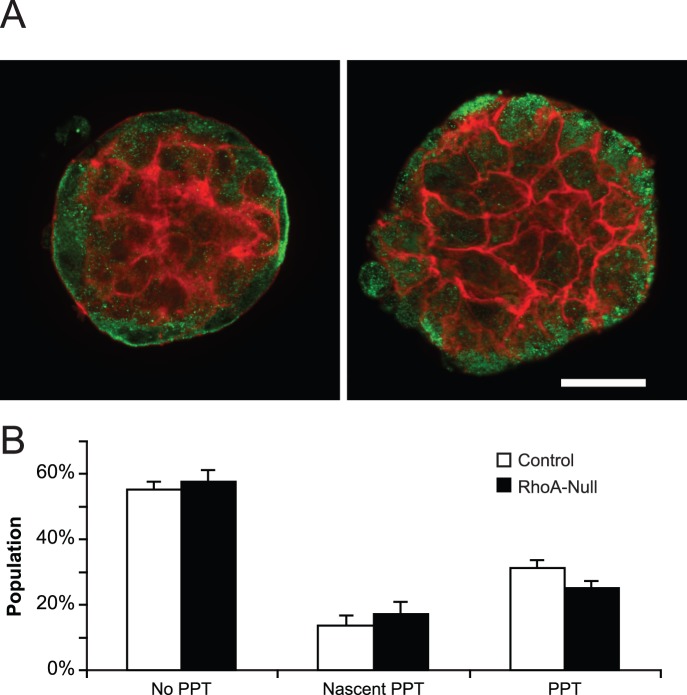
Microscopy shows that RhoA-null megakaryocytes have normal cytoskeleton architecture and do not increase proplatelet number. (A) Shown are representative laser confocal images of megakaryocytes (control and RhoA-null, left and right respectively) stained for β1-tubulin (antibody, green) and F-actin (phalloidin, red). Magnification is 63× and the scale bar is 20 um. (B) Megakaryocytes were imaged by phase contrast microscopy, and they were characterized on the basis of exhibiting proplatelets, nascent proplatelets (∼ <2 um), or no proplatelets. Mean ±SE is shown. N = 3.

Confocal microscopy of the cytoskeletal architecture of RhoA-null megakaryocytes stained for β1-tubulin and F-actin did not reveal any detectable abnormalities (Figure 4A). Expression patterns of these two molecules was similar in both RhoA-null and control megakaryocytes. As expected, β1-tubulin was localized at the edge of both megakaryocytes and proplatelets. F-actin expression was found predominately in the cortex, occasionally in the periphery, and at times, actin was even extended out beyond the β1-tubulin marginal band. Previous studies demonstrated that dominant negative forms of RhoA can increase proplatelet length, which argued that RhoA might contribute to the regulation of proplatelet formation [3]. However, we did not observe any defect in RhoA-null megakaryocytes projecting proplatelets (Figure 4B).

**Figure 5:**
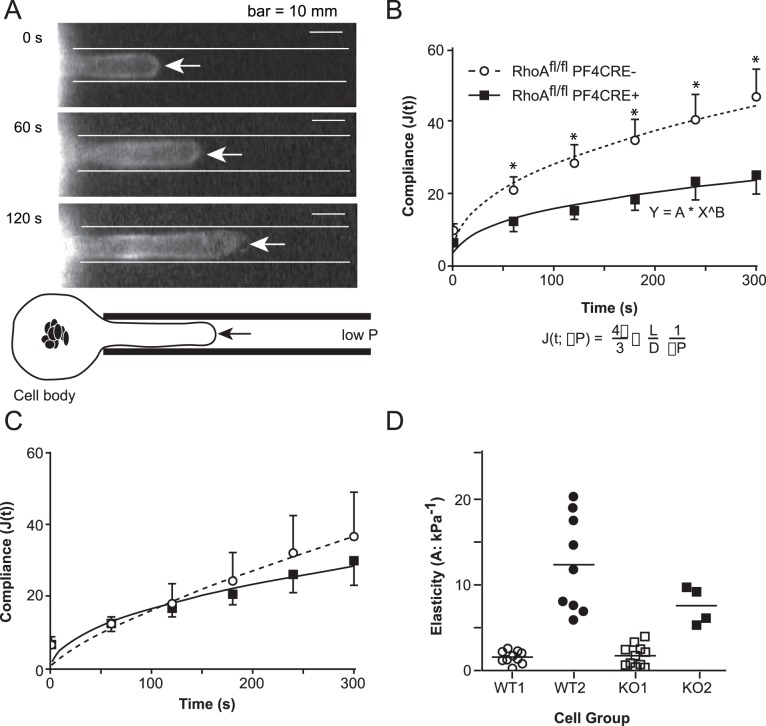
Large RhoA-null megakaryocytes are less compliant than normal sized megakaryocytes. (A) Pictured are representative cells being aspirated with micropipettes (ΔP 0.3–5.7 kPa) causing the cell membrane protrusion (arrows) to extend in length over time. Compliance (J) was computed from the length of the protrusion, and plotted as a function of time. This data exhibited power law behavior: J(t) = A*(x/x_0_)∧B, where A determines elasticity and B determines rheostatic properties. (B) When the data was not normalized by size, RhoA^fl/fl^ PF4CRE^+^ derived megakaryocytes were less compliant than the control RhoA^fl/fl^ PF4CRE^−^ derived megakaryocytes at all times tested. Paired t-test analysis demonstrated that P<0.05 for time points indicated by a "*". (C) When only similarly sized megakaryocytes were compared (large controls vs. KO), RhoA-null megakaryocytes had similar compliance curves to that of their controls. Control RhoA^fl/fl^ PF4CRE^−^: n = 25. RhoA^fl/fl^ PF4CRE^+^: n = 22. Shown is the mean ± standard deviation for three independent experiments. Paired t-test analysis demonstrated that P>0.05 at all analyzed time points. (D) The basis for normalizing elasticity with size was established as control megakaryocyte elasticity was bimodally distributed, segregating into two groups, WT1 and WT2. Based upon the WT threshold criterion for elasticity, RhoA-null megakaryocytes (KO) were segregated into two groups, KO1 and KO2. The differences in membrane compliance can be entirely accountable by the differences in the sizes of wild type and mutant megakaryocytes.

To test whether genetically ablating RhoA perturbs megakaryocyte membrane rheology and alters thrombopoiesis, we measured cortical tension and compliance of the megakaryocytes by using micropipette aspiration to deform the cell membrane (Figure 5A) [26]. This biophysical model approximates the shear forces within the bone marrow sinusoid capillaries that help drive proplatelet formation. We found that RhoA-null megakaryocytes had stiffer membranes that prevented them from deforming upon aspiration (Figure 5B). This demonstrates that the RhoA-null megakaryocytes, as a group, had a lower compliance rate and a higher cortical tension than their controls. However, when factoring for size, the compliance curves of RhoA-null megakaryocytes were similar to the control group (Figure 5C). Hence, we observed that compliance correlated with size (Figure 5D). Altogether, these data show that there is not a significant difference in deformability of equally size wild type and mutant megakaryocytes. The differences in compliance observed were entirely due to their higher ploidy and larger size of RhoA-null megakaryocytes. This is consistent with previous publications that demonstrated that specific cytoskeletal proteins start to accumulate as megakaryocytes increase in size and undergo endomitosis [26]. Thus, our data suggests that RhoA-null megakaryocyte proplatelets may fragment more quickly because of their diminished compliance.

### *In vivo* Platelet Production is Impaired by the RhoA Mutation

**Figure 6:**
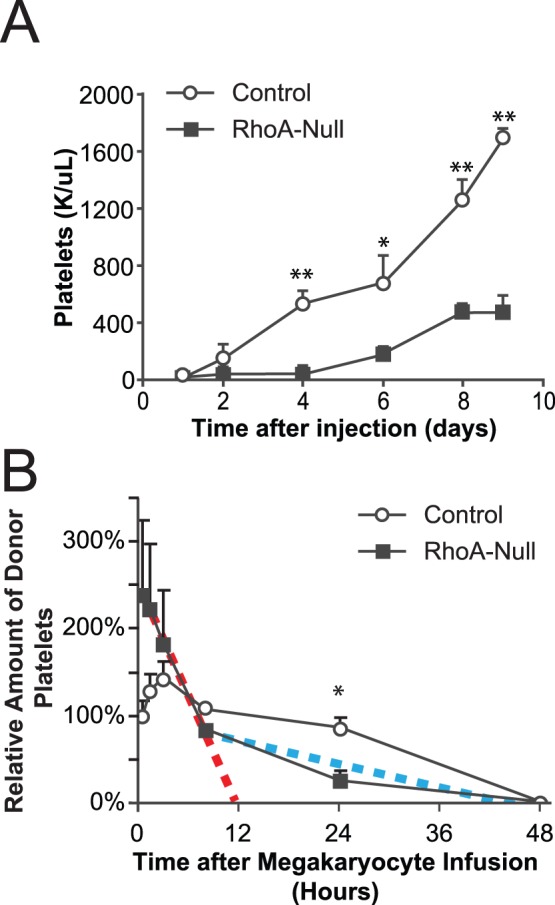
Platelet production in RhoA-null megakaryocytes is impaired. (A) Mice were injected with GPIbα antibodies, and platelet levels were counted manually from blood smears. While the control mice began to recover from the depletion after two days, RhoA^fl/fl^ PF4CRE^+^ mice did not begin to recover until after four days. Mean ±SE is shown; * indicates P<0.05; ** indicates P<0.005. N = 5. (B) The platelet survival of infusing either RhoA-null megakaryocytes or controls into the mαIIb^−/−/^hαIIb^+/+^ mice was measured. Platelet production of the infused RhoA-null megakaryocytes diminished over a 24-hour period of time as compared to the controls (* indicates P<0.005). The mean ±SE is shown. N = 5. The red line shows the “best fit” trajectory of platelet depletion during the first eight hours, while the blue line approximates the platelet clearance rate from eight hours onward.

To determine whether this change in membrane rheology affects platelet production, we immunodepleted platelets from RhoA^fl/fl^ PF4CRE^+^ mice and control mice using antibodies directed against murine GPIbα. Platelets were rapidly cleared from the circulation, decreasing to 20–25% of the baseline platelet counts within three hours post-injection. The platelet counts in the control mice began to increase within the first two days after the platelet depletion (Figure 6A). However, platelet recovery in RhoA^fl/fl^ PF4CRE^+^ mice was delayed, and recovery did not begin until after four days had elapsed following the injection of the platelet depleting antibody. This demonstrates that platelet biogenesis *in vivo* is impaired by the loss of RhoA.

The impaired platelet production *in vivo* could be attributable to a defect in platelet production by megakaryocytes, or by the lower number of megakaryocytes found in RhoA^fl/fl^ PF4CRE^+^ mice. To address this issue, we analyzed platelet production kinetics *in vivo* by infusing mature megakaryocytes into the retro-orbital sinuses of recipient transgenic hαIIb^+/+^/mαIIb^−/−^ mice described earlier [17]. This technique allowed us to inject an equal number of megakaryocytes derived from RhoA^fl/fl^ PF4CRE^+^ mice and RhoA^fl/fl^ PF4CRE^−^ mice, thereby eliminating the possibility that any differences observed were due to the defective number of megakaryocytes seen in RhoA^fl/fl^ PF4CRE^+^ mice. RhoA-null megakaryocytes rapidly released platelets, but after 24 hours, the platelet levels were significantly decreased to less than 10% of the initial reading (Figure 6B). In marked contrast, the platelets produced in the littermate controls more slowly, but lasted longer. The platelets derived from normal megakaryocytes did not start to disappear from the circulation for the first 24 hours after the initial release. Analyzing the kinetics of the longevity of platelets released from RhoA-null megakaryocytes demonstrated two populations of platelets were cleared at different rates from the circulation. While the majority of RhoA-null platelets cleared rapidly (red line), there was a small subpopulation that had a longer lifespan (blue line). These longer lasting RhoA-null platelets had a lifespan that was comparable to the lifespan of the control platelets. This is suggestive of a selection process that may allow this longer-living subpopulation of platelets to predominate in the circulation.

## Discussion

We observed that RhoA^fl/fl^ PF4CRE+ mice have macrothrombocytopenia, shortened megakaryocyte survival, and decreased platelet production. This demonstrates that RhoA plays a critical role in platelet development. Infusion experiments showed that RhoA-null megakaryocytes tend to release platelets quickly and decay faster than normal. We hypothesize that the cause of the accelerated proplatelet fragmentation and platelet release in RhoA^fl/fl^ PF4CRE+ mice is due to the larger size and ploidy of megakaryocytes, and abnormal cell membrane compliance, which are induced by the loss of RhoA. Our working model is consistent with the proposal that RhoA is involved in multiple steps of megakaryocyte development. This includes cytokinesis during the promegakaryoblast stage, endomitosis, migration within the bone marrow matrix, and proplatelet formation.

### Regulation of Megakaryopoiesis by RhoA

Macrothrombocytopenia, increased megakaryocyte ploidy, and a decreased number of megakaryocytes in the bone marrow of RhoA^fl/fl^ PF4CRE^+^ mice all point to a significant role for RhoA in megakaryocyte development. During megakaryopoiesis, the actin contractile ring is speculated to be regulated by several known effectors of RhoA, including ROCK and citron kinase, which generate the contractile forces [27]. In addition, mDia, formin, and phosphatidylinositol 5-kinase (PIP5K) are other known RhoA effectors that contribute to the assembly of the actin ring [10]. However, during endomitosis, RhoA is absent in the cleavage furrow, thereby down-regulating RhoA effectors, which in turn disrupts the formation of the contractile ring. Two mitotic RhoA regulators, Guanine Exchange Factors (GEFs) such as H1 and ECT2, have been shown to coordinate RhoA translocation to the contractile ring [5]. The expression of these GEFs is lower in cells with higher ploidy, and polyploidization is decreased with the ectopic expression of these RhoA GEFs. Additionally, *in vitro* experiments with cultured megakaryocytes treated with the pharmacological inhibition of RhoA by the use of C3 transferase induce higher cell ploidy, presumably by accelerating the reversal of cytokinesis [4], [28]. Similar outcomes result from the use of inhibitors of RhoA effectors and downstream signaling pathways such as the use of blebbistatin against myosin or Y27632 against ROCK [23], [29]. Our data provides genetic evidence that supports these pharmacologic and overexpression experiments. Together with the published work, our experiments now definitively identify RhoA as a multifunctional regulator of developing megakaryocytes and platelets [4], [5], [23], [28], [29].

### Platelet Production in RhoA-null Megakaryocytes

Previously, pharmacological inhibition of RhoA caused increased proplatelet production. However, we observed normal proplatelet formation in our genetically deleted RhoA-null megakaryocytes. It is unclear why there is a distinction between the two approaches of impairment given that the target protein is the same. Perhaps, the difference is developmental, where long-term ablation of RhoA through genetic manipulation causes changes in the cytoskeleton (although permissive for premature proplatelet formation), whereas the cytoskeleton is not affected by the shorter duration of treatment by the pharmacological inhibitor. An alternative explanation for the different results obtained with pharmacologic inhibitors and genetically engineered mice is that the results observed following pharmacologic inhibition of RhoA might be attributable to off-target inhibition of other Rho-family GTPases.

Because RhoA regulates actin stress fiber and focal adhesion formation, we postulated that the impairment of the integrity of these actin-rich structures would decrease membrane compliance, while increasing proplatelet formation [30]. However, we discovered that the disruption of RhoA caused an increase in cortical tension and a decrease in compliance. A more detailed analysis demonstrated that this is attributable to the larger size of RhoA-null megakaryocytes since membrane cortical tension varies with size.

The increase in membrane stiffness and fragmentability may impair the repartitioning of demarcation membranes leading to accelerated platelet release. When we measured platelet production by infusing megakaryocytes, we observed that platelets were rapidly released, although only 10% of the platelets survived 24 hours post-infusion. As we and Pleines et al. have observed, circulating RhoA-null platelets have only a moderate deficit in lifespan that is certainly not sufficiently shortened to explain the magnitude of thrombocytopenia observed *in vivo* in RhoA^fl/fl^ PF4CRE+ mice. Nor could this account for the precipitous drop in platelet levels during the megakaryocyte infusion experiments [8]. Furthermore, the decrease in the persistently circulating platelets derived from infused RhoA-null megakaryocytes was not due to the shortened survival of the infused megakaryocytes, since these cells become quickly embedded in the pulmonary capillary beds and released their platelets within three hours [17].

Insight into the apparent discrepancy in RhoA^fl/fl^ PF4CRE+ platelet survival is derived from mice that lack filamin A in their platelets. These mice have a similar macrothrombocytopenia [31]. They have large, fragile platelets that are generated prematurely and rapidly removed from circulation by macrophages. We speculate that the rapid premature release of platelets in RhoA^fl/fl^ PF4CRE^+^ mice promotes the formation of large, fragile platelets that are also rapidly cleared due to hemodynamic stresses. In addition, the lack of RhoA in platelets may alter the anchoring of the cell membrane to the cytoskeleton, accelerating their shortened survival. Megakaryocyte infusion data (Figure 5B) shows a steep decline of platelet levels, thereby demonstrating that some nascent RhoA-null platelets are inherently more susceptible to early clearance. As shown by the biphasic decay rates, there remains a subpopulation of platelets that can survive longer, and these are likely to be the “survivors” that predominate in the circulating platelet pool in RhoA^fl/fl^ PF4CRE^+^ mice. This would explain the reason why the lifespan of circulating RhoA-null platelets was similar to their controls in the platelet half-life experiment. In those experiments, we were only analyzing the circulating (longer surviving) population of RhoA-null platelets.

### RhoA-mediated Stress Fiber Regulation of Proplatelet Extension

Notably, the macrothrombocytopenia phenotype found in RhoA^fl/fl^ PF4CRE+ mice is similar to that found in mice with filamin A-null, GPIbα-null, GPIbβ-null, or Myh9-null megakaryocytes [31]–[33]. Perhaps the shared phenotype is due to a final common pathway. This suggests that megakaryocyte development requires RhoA to generate stress fibers that are cross-linked with filamin A and the GPIb-IX-V VWF receptor complex [34]. This has two implications. First, RhoA-induced stress fibers anchor to filamin A and the receptor complex to maintain cell membrane structural integrity within the megakaryocyte and within the platelet. Second, RhoA-induced stress fibers couple with this complex to serve as a mechanosensor to guide the proplatelets during migration within the bone marrow. Like the VWF receptor complex, the integrin αIIbβ3 fibrinogen receptor may also transduce “outside-in” pathways. Thus, RhoA could serve as a link regulating proplatelet formation via integrins, in addition to VWF [35].

In summary, we have demonstrated a role for RhoA in megakaryopoiesis and in proplatelet formation *in vivo* that is attributable to effects of RhoA at many stages of megakaryocyte development. Loss of RhoA affects megakaryocyte ploidy and size, as well as membrane rheology. This results in the aberrant release of large platelets that are rapidly cleared from the blood circulation.
